# Life cycle assessment of iron-biomass supported catalyst for Fischer Tropsch synthesis

**DOI:** 10.3389/fchem.2024.1374739

**Published:** 2024-03-27

**Authors:** Muhammad Amin, Hamad Hussain Shah, Abdul Basit Naveed, Amjad Iqbal, Yaser Gamil, Taoufik Najeh

**Affiliations:** ^1^ Interdisciplinary Research Center for Hydrogen Technologies and Carbon Management (IRC-HTCM), King Fahd University of Petroleum and Minerals (KFUPM), Dhahran, Saudi Arabia; ^2^ Department of Engineering, University of Sannio, Benevento, Italy; ^3^ Department of Chemistry, University of Louisville, Louisville, KY, United States; ^4^ Faculty of Materials Engineering, Silesian University of Technology, Gliwice, Poland; ^5^ Department of Civil Engineering, School of Engineering, Monash University Malaysia, Subang Jaya, Selangor, Malaysia; ^6^ Operation and Maintenance, Operation, Maintenance and Acoustic, Department of Civil, Environmental and Natural Resources Engineering, Lulea University of Technology, Luleå, Sweden

**Keywords:** biomass, life cycle assessment, Fischer Tropsch synthesis, catalyst, environmental impact

## Abstract

The iron-based biomass-supported catalyst has been used for Fischer-Tropsch synthesis (FTS). However, there is no study regarding the life cycle assessment (LCA) of biomass-supported iron catalysts published in the literature. This study discusses a biomass-supported iron catalyst’s LCA for the conversion of syngas into a liquid fuel product. The waste biomass is one of the source of activated carbon (AC), and it has been used as a support for the catalyst. The FTS reactions are carried out in the fixed-bed reactor at low or high temperatures. The use of promoters in the preparation of catalysts usually enhances C_5+_ production. In this study, the collection of precise data from on-site laboratory conditions is of utmost importance to ensure the credibility and validity of the study’s outcomes. The environmental impact assessment modeling was carried out using the OpenLCA 1.10.3 software. The LCA results reveals that the synthesis process of iron-based biomass supported catalyst yields a total impact score in terms of global warming potential (GWP) of 1.235E + 01 kg CO_2_ equivalent. Within this process, the AC stage contributes 52% to the overall GWP, while the preparation stage for the catalyst precursor contributes 48%. The comprehensive evaluation of the iron-based biomass supported catalyst’s impact score in terms of human toxicity reveals a total score of 1.98E−02 kg 1,4-dichlorobenzene (1,4-DB) equivalent.

## 1 Introduction

Fischer-Tropsch synthesis (FTS) is a process that has been used for the conversion of syngas (a mixture of hydrogen and carbon monoxide) into liquid fuel hydrocarbons (Chernyak et al., 2020); (Munir et al., 2023). A series of catalysts have been used for this purpose. The most used catalysts are iron, cobalt, nickel, and ruthenium. Among all these catalysts, iron catalysts are cheap and provide more C_5+_ product distribution. Recently, the research has shifted toward the iron-biomass supported catalyst. Because the biomass support used for this purpose is produced from a waste biomass source, support has a lot of advantages over catalytic performance (Amin et al., 2022a).

Naturally, a wide range of biomass sources are available for activated carbon (AC) production. When using AC as a carbon support, several aspects must be addressed, the most significant of which are the surface area of the AC, its composition, and the activation agent used to prepare the AC (Amin et al., 2022b). The greater the surface area, the higher the gas retention period, which improves catalytic activity and selectivity for liquid fuel products (Amin and Shah, 2022); (Amin, 2023). The composition of AC has a direct influence on catalyst performance; for example, the carbon support generated from the biomass source “Lantana Camara” contains certain oily compounds by nature (Sousa et al., 2010)–(Kumar et al., 2020). Finally, the activation agent is regarded as one of the most significant aspects in catalyst preparation. Many activation agents, including H_3_PO_4_, KOH, ZnCl_2_, FeCl_3_, H_2_SO_4_, HCL, K_2_CO_3_, and others, have been used in the AC production. Some activation agents, such as KOH, aid in the creation of potassium-based catalysts. Potassium acts as a promoter.

The formation of catalysts through synthesis procedures results in the generation of residual byproducts, primarily consisting of liquid residues, flue gases, and a relatively small quantity of solid waste. The environmental consequences associated with these processes are greatly influenced by the specific synthesis method employed and the initial materials used. In most conventional thermochemical approaches used to synthesize catalysts, it is necessary to prepare an initial solution containing a minimal concentration of dissolved chemicals. To illustrate, recently researcher conducted the Cu/AC catalysts synthesis, supported on AC through an impregnation process. In this specific case, the initial solution employed contained Cu(NO_3_)_2_ at a 0.001 mol L^−1^ concentration (Abdedayem et al., 2015). In another study, catalysts composed of Ni/Al_2_O_3_ were synthesized using a co-precipitation technique. Notably, the starting solution for this process consisted of Al(NO_3_)_3_ and Ni (NO_3_)_2_ at concentrations of 0.098 mol L^−1^ and 10.085 mol L^−1^, respectively (Jung et al., 2012). Hence, to produce 1 kg of catalyst, a substantial quantity of initial solution is typically necessary, and this solution often turns into waste following its application. The process of oxidative calcination of catalyst supports impregnated with metal salts, particularly nitrates, results in the release of NO_2_ gas. Additionally, when metal nitrate and/or chloride mixtures are subjected to hydrogen reduction instead of calcination, it results in the generation of waste acid solutions.

Life cycle assessment (LCA) is a commonly employed method for conducting a thorough assessment of the environmental effects of processes across their entire life cycle (Agarski et al., 2017)–(Al-Mawali et al., 2021). However, there has been limited LCAs studies conducted on catalyst synthesis procedures. For instance, evaluating the emissions of atmospheric pollutants associated with the catalysts employed in the hydrotreating of bio-oil derived from fast pyrolysis through LCA (Snowden-Swan et al., 2016). The study finds significant differences in GHG emissions depending on the catalyst used, with NiMo/Al_2_O_3_ estimated at 5.5 kg CO_2_-e/kg catalyst and Ru/C varying significantly between 80.4 kg CO_2_-e/kg (economic allocation) and 13.7 kg CO_2_-e/kg (mass allocation). In another study, the cradle-to-gate LCA framework was applied for the comparative analysis of potential water treatment methodologies, which involved the use of two different types of carbon nanofiber-supported catalyst (Yaseneva et al., 2014). The results showed that the potential water purification process based on catalytic reduction of bromates, the Pd/CNF/SMF catalyst is considerably more efficient in terms of activity, cost and environmental impact. A study examining the environmental effects associated with producing fine chemicals through TiO_2_ (solar) photocatalysis was performed using LCA. This study included a comparative analysis with similar reactions performed under thermal conditions (Ravelli et al., 2010). The authors employed three assessment methods: LCA, EATOS, and EcoScale, highlighting the environmental trade-offs between photocatalytic and thermal processes. The findings indicated that thermal reactions generally have a lower environmental impact compared to photocatalytic methods, primarily due to the high solvent usage in photocatalytic reactions. However, photocatalytic reactions offer higher yields and are marked as potentially “cleaner” processes, suggesting that with optimization, such as solvent recovery or concentration adjustments, photocatalysis could become a more environmentally friendly synthetic strategy. Additionally, Fernandez et al. (2016) utilized LCA to assess enzyme-catalyzed processes employed in biodiesel production and their implications on the environment (Peñarrubia Fernandez et al., 2017). The study results showed that using waste cooking oil as a feedstock significantly reduces environmental impacts, highlighting the enzyme-catalyzed process’s potential for lower energy and chemical usage. The study emphasizes the importance of feedstock selection and process technology in minimizing the environmental footprint of biodiesel production, demonstrating considerable environmental benefits when utilizing waste cooking oil and enzyme-catalyzed processes. A comparative LCA to evaluate the treatment of pharmaceutical wastewater using heterogeneous catalysts is performed by (Rodríguez et al., 2016). The study uses ReCiPe and IPCC 2007 methodologies for evaluating environmental impacts, introducing the Water Footprint as a novel comparison tool. Results indicate the heterogeneous Fenton process is more environmentally friendly, reducing the water footprint by over 77% compared to the homogeneous process. Key findings highlight the significant environmental impact of sludge recovery in the homogeneous process and recommend for the heterogeneous approach as a sustainable alternative for wastewater treatment. Recognizing the significant variations in properties and applications among different catalysts, this study focuses on conducting LCA for the synthesis processes of iron-based biomass supported catalyst. Based on the outcomes of prior studies focusing on the synthesis of catalysts, the authors identified a gap in existing literature, noting the absence of LCAs specifically focused on the synthesis processes of iron-based biomass-supported catalysts. The novel process for synthesis of iron-based biomass supported catalyst developed by the authors and thoroughly evaluated and discussed the environmental implications of this novel process.

## 2 Material and methods

Waste tree leaves from any tree are necessary for carbon support. Lantana camara tree leaves were obtained for this investigation. A washing step was necessary as the fallen tree leaves contained some dust particles. Following washing, the waste biomass was dried in an open environment to eliminate moisture. The dried waste biomass source was crushed and grinded and passed through a sieve of 150–250 µm. The crushing and grinding steps are considered very important because they are responsible for the higher surface area of the AC. In the next step, the absorption of the activation agent was performed (Amin et al., 2022a).

The selection of the activation agent is based on the promoter, hemicellulose, and lignin content of the waste biomass source. For example, if potassium is used as a promoter, then K_2_CO_3_ is preferred as an activation agent. A promoter’s function is to increase the rate of a chemical reaction. Therefore, in the study, potassium was used as a promoter. After absorption of the biomass in the activation agent, the sample was calcined in the furnace at a high temperature (500°C–600°C) (Munir et al., 2023). Finally, the prepared char was washed with HCl or distilled water to remove the excess amount of activation agent. Finally, the highly surface area-based AC was ready to be used in the catalyst as a carbon support (Amin et al., 2022c). In the next step, the prepared catalyst was mixed with the iron nitrate. For proper absorption of iron and AC, the sample must be mixed by using a magnetic stirrer for 1 day. Therefore, the sample was mixed for 1 day. The sample was calcined at 350°C to obtain the iron-supported biomass catalyst. The next was the addition of a promoter. Potassium was used as a promoter. The potassium has a very low melting point and completely reacts with the iron-biomass catalyst at 400°C. The prepared Fe-C-K was ready for use in the FTS reactor. In the fixed bed reactor, the reaction was carried out for the FTS process. In the fixed bed reactor, 1 g of prepared catalyst was loaded and it was reduced at 450°C for 5 h by using hydrogen gas at a flow rate of 30 sccm. For inert gas, nitrogen was used at a flow rate of 10 sccm. The reactor was cooled to 350°C to promote the reaction at 20 bar in the presence of a H_2_/CO (1:1 ratio). To achieve equilibrium, a back pressure valve was used. The reaction was carried out for 10 h, and the liquid product was collected from the separator. The liquid product was analyzed with the help of GC-MS, and the gas was analyzed by using a GC machine (Amin et al., 2022a).

The process estimation calculations are based on the major energy consumption involved in the preparation of catalyst. For this purpose, the major catalyst preparation steps are washing of biomass (biomass was washed using filter paper process), grinding and sieving, chemical activation, and oven drying. For grinding biomass, a juicer grinder was used. For the chemical activation, a muffle furnace was used. Finally, the catalyst was dried in the oven. As the catalyst was prepared on a lab scale, the biomass collection, biomass washing with water, and chemical transportation required no electricity. The process parameters are shown in Table 1.

**TABLE 1:** The electricity consumption values of the process.

Process	Time (hours)	Type of sources	Machine	Input amount	References
Washing Biomass	1	Water	Filter Paper	900 mL	(Amin et al., 2022b), (Amin et al., 2022c)
Grinding/sieving	2	Electricity	Juicer Grinder	0.3 kWh	
Chemical Activation	3	Electricity Distilled Water	Furnace (4 kWh)	12 kWh 5.156 mL	
Washing/Oven Drying	6	Electricity Water	Oven (1 kWh)	6.0 kWh 1851 mL	

### 2.1 Life cycle assessment

**FIGURE 1:**
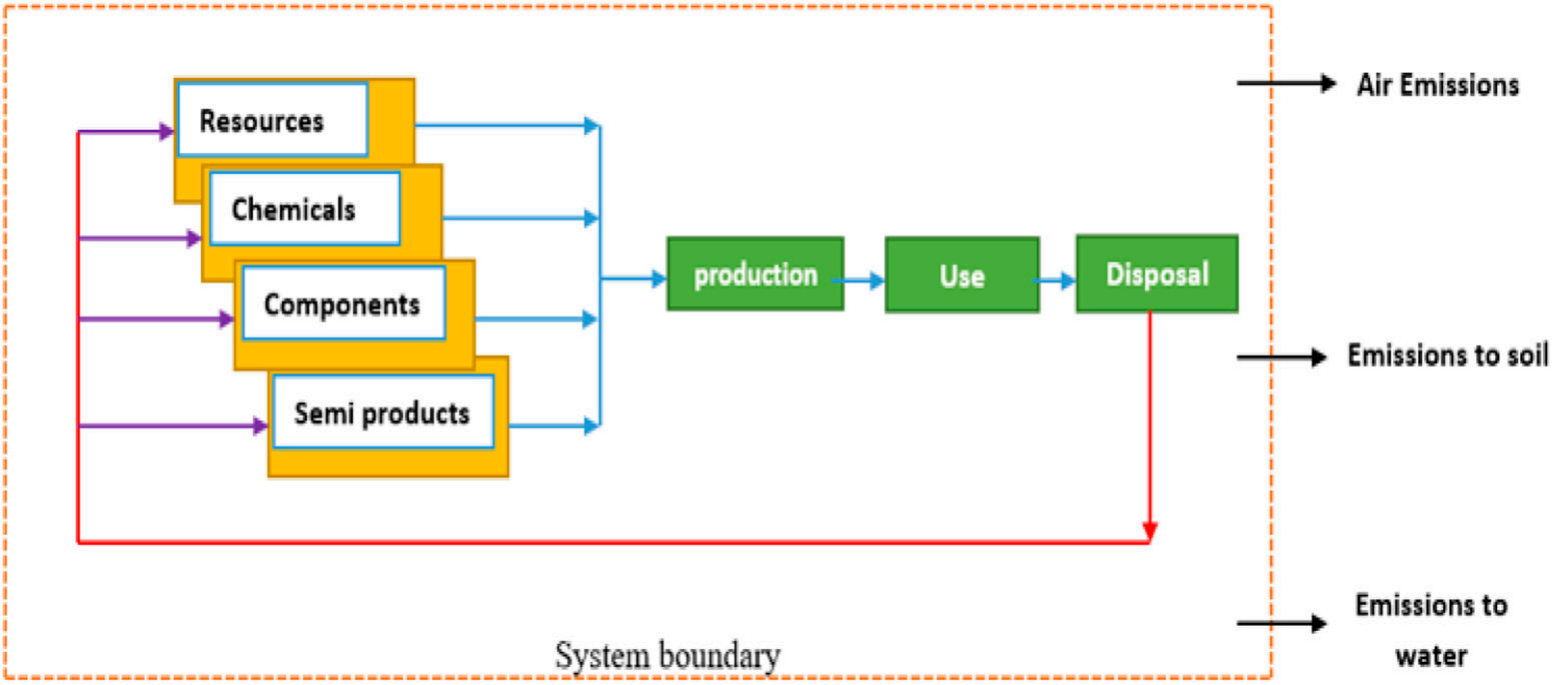
Life cycle of a product or process within the system boundary.

**FIGURE 2:**
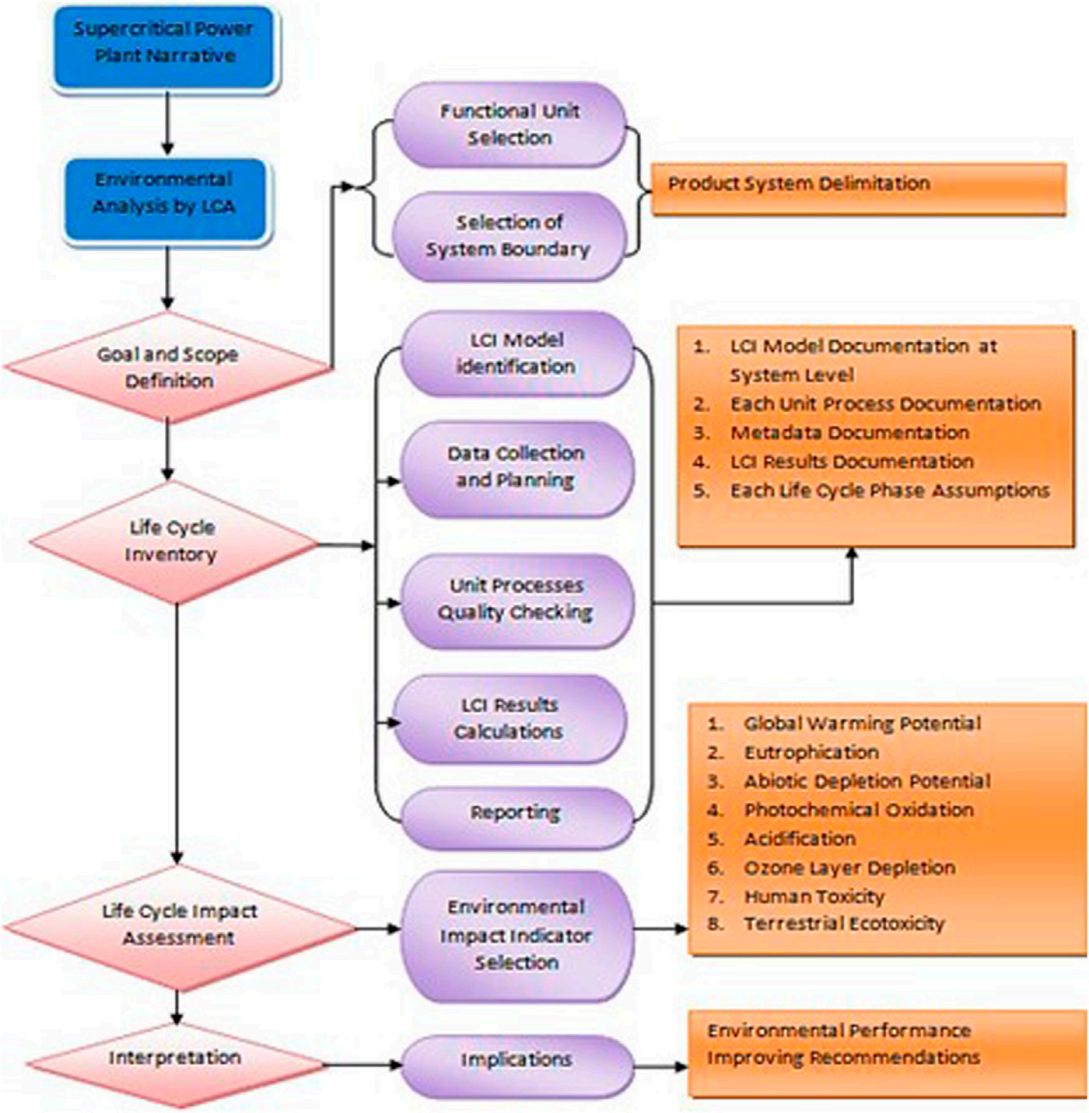
Life cycle assessment framework.

The LCA methodology was developed as a comprehensive tool for evaluating the environmental impacts associated with products or services. To perform its intended function, a product undergoes various stages such as development, manufacturing, distribution, use, maintenance, and end-of-life management. The production of the product necessitates several supportive functions, including resource extraction and conversion into materials, components, or supplementary substances. Moreover, the infrastructure plays a pivotal role in providing indispensable support to the manufacturing facility and its workforce. The transportation processes form a critical physical link between these activities. It is important to acknowledge that all of these supporting activities entail the consumption of resources and generate environmental impacts. To obtain a comprehensive understanding of the overall environmental repercussions stemming from the product or service, the analysis must, therefore, focus on the product system or its life cycle, as depicted in Figure 1 in a general form. The ISO 14000 series of standards for Environmental Management introduced four distinct standards for life cycle assessment and its primary stages (Figure 2) (ISO - ISO 14040, 2006); (ISO, 2006); (Guinée, 2002); (Guince, 2018).

### 2.2 Goal and scope

**FIGURE 3:**
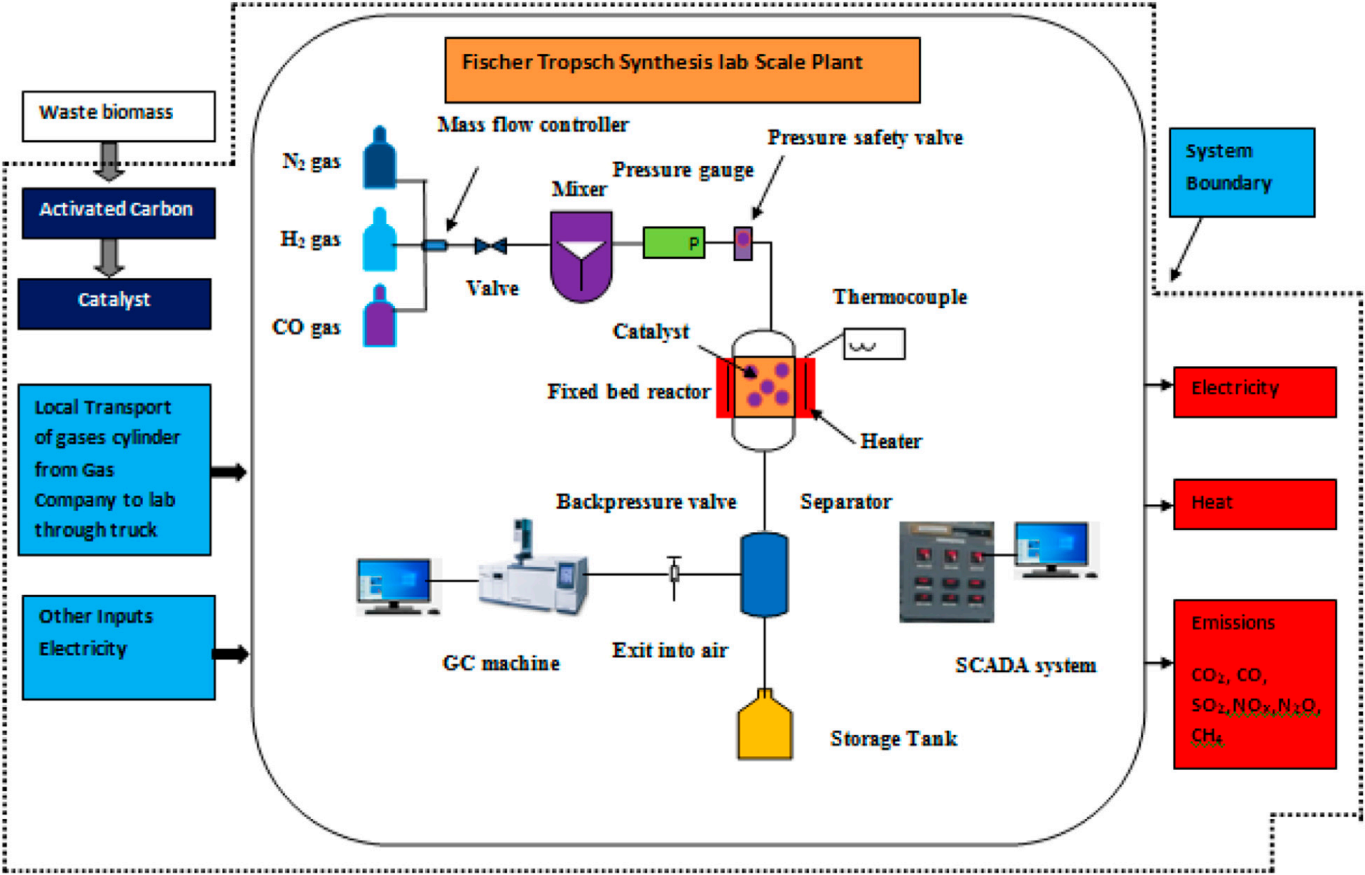
System boundary of the present study.

The aim of this assessment is to conduct a comprehensive LCA of the synthesis process for an iron-based biomass supported catalyst and evaluate its environmental impacts. The scope of this analysis encompasses various stages, including raw material extraction from the environment, semi-product production, and the synthesis of the iron-based biomass supported catalyst, as illustrated in Figure 3. However, certain aspects such as the transportation of raw materials, the utilization phase of the catalyst, and the management of end-of-life waste fall outside the defined system boundaries for this particular analysis. In LCA, the concept of a functional unit is employed to facilitate the comparison of products or processes that serve similar functions. Within this specific study, the functional unit is defined as the production of 1 g of the iron-based catalyst. This standardizes the assessment and allows for meaningful comparisons to be made between different catalyst production processes or alternative catalyst products.

In order to determine the waste outputs per 1 g of synthesized catalyst, the catalyst was prepared within our laboratory setting. Under these experimental conditions, it is assumed that the generated liquid waste is directed towards the sewage system for disposal, while the gaseous emissions are released into the atmosphere, and any solid waste is appropriately deposited into designated landfills.

In the conventional impregnation process, it is customary for the metal salt starting solution to undergo a filtration step, which consequently generates a waste solution unless the liquid phase is subjected to evaporation to minimize waste generation. Filtration generates solid waste in the form of used filter paper. During the synthesis process, precursor mixtures containing metal salts on catalyst supports are commonly subjected to calcination in an air atmosphere, leading to the formation of metal oxides from the salts. When employing metal nitrates as the starting materials, the resulting flue gas primarily comprises NO_2_ diluted in the surrounding air. On the other hand, if the precursor mixtures are subjected to direct reduction within a hydrogen flow instead of undergoing calcination, the metal salts are transformed into metallic forms, thereby leading to the generation of waste acid solutions. The specific type of acid formed (HCl, HNO_3_, or a mixture of these acids) depends on the metal salt employed in the synthesis process. Subsequently, these gases containing acids are directed through a wash bottle, facilitating their collection in the form of diluted acids.

### 2.3 Life cycle inventory

In this study, two processes are considered. First process consists of AC production and second process consists of catalyst preparation. The foreground data for both processes were acquired through on-site experiments conducted under laboratory conditions. To complement any missing foreground and background data, the ecoinvent database was utilized. The environmental impact assessment modeling was carried out using the OpenLCA 1.10.3 software. In this study, the collection of precise data from on-site laboratory conditions is of utmost importance to ensure the credibility and validity of the study’s outcomes. By integrating experimental data obtained directly from the laboratory and leveraging external databases like ecoinvent, a thorough assessment of the environmental impacts is performed. The utilization of advanced software tools such as OpenLCA enables the comprehensive analysis and evaluation of these environmental impacts, thereby enhancing the overall robustness of the research findings. Table 2 shows the life cycle inventory of both the process.

**TABLE 2:** Life cycle inventory data as per functional unit.

Parameters	Unit	Processes	
		Activated carbon production	Catalyst preparation
*Background data*			
Inputs			
Lantana Camara	g	24.00	—
H_3_PO_4_	g	12.13	—
Activated carbon	g	—	4.00
Electricity	kWh	18.33	24.00
water	g	11,312	20.00
Filter paper	—	8	—
Iron Nitrate Fe(NO_3_)_3_.9H_2_O	g	—	3.20
Potassium Carbonate (K_2_CO_3_)	g	—	0.26
*Foreground data*			
Outputs			
Activated carbon	g	4.00	—
Fe-C (catalyst)	g	—	1.00
H_2_	g	10.00	12.10
CO	g	127	132
CO_2_	g	107	109
CH_4_	g	219	215
NO_2_	g	5.31	11.27
wastewater	g	8,600	12.45

### 2.4 Impact assessment

Following the compilation of comprehensive data for the life cycle inventory, a thorough life cycle impact assessment can be conducted to evaluate the environmental implications of iron-based biomass catalyst synthesis processes. The CML-IA baseline method, a widely employed approach in environmental impact assessments, was employed for this purpose. It is developed by the Institute of Environmental Sciences (CML) at Leiden University. It is designed to quantify and assess the environmental impacts of products and processes throughout their entire life cycle, from raw material extraction through manufacturing, use, and disposal. The CML-IA baseline method is obtained by applying specific impact assessment categories and characterization factors to convert LCI data into indicators of potential environmental impacts. These categories typically include climate change (global warming potential), ozone depletion, human toxicity, ecotoxicity, photochemical oxidant formation, acidification, eutrophication, and resource depletion, etc. The CML-IA baseline method is characterized by its focus on midpoint impact categories, providing detailed insight into specific types of environmental impacts. This approach helps decision-makers understand the direct effects of emissions and resource use, facilitating targeted strategies for environmental improvement. In order to focus on the most significant impacts, a selection of five impact categories has been made, namely global warming, human toxicity, photochemical oxidation, eutrophication, and acidification. By excluding impact categories with contributions below 1 percent, which can be considered statistically insignificant compared to the impacts of other categories, the focus is directed towards the most substantial environmental concerns. Thus, the excluded impact categories encompass Marine aquatic ecotoxicity, Ozone layer depletion, Freshwater aquatic ecotoxicity, Abiotic depletion, and Terrestrial ecotoxicity. Further investigation and analysis of these prominent impact categories will yield valuable insights into the ecological consequences associated with iron-based biomass catalyst synthesis processes. This strategic exclusion enables efficient allocation of resources and targeted mitigation efforts to effectively reduce the overall environmental footprint of the catalyst synthesis processes.

## 3 Results and discussion

### 3.1 Interpretation of environmental impacts

**FIGURE 4:**
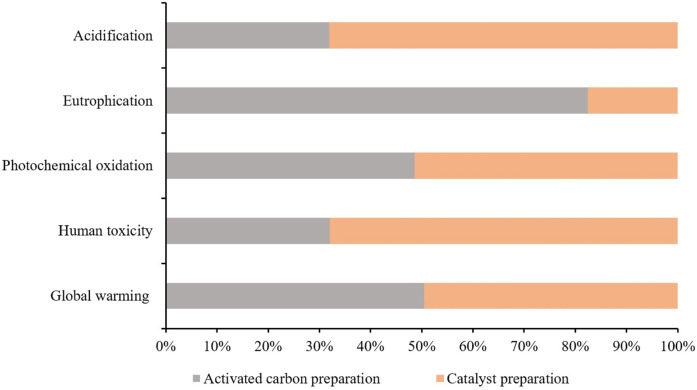
Two stage relative contribution of iron-based biomass catalyst synthesis process.

Table 3 presents the characterized results obtained from iron-based biomass catalyst synthesis processes. These processes were investigated and evaluated in terms of their respective performances and characteristics. Additionally, Figure 4 illustrates the two-stage relative contribution of the iron-based biomass catalyst synthesis process in terms of global warming, human toxicity, photochemical oxidation, eutrophication, and acidification. In this section, a comprehensive analysis of the results pertaining to the iron-based biomass catalyst synthesis processes is presented. The obtained data and outcomes from each synthesis method are thoroughly elucidated and discussed in detail. The findings encompass various performance metrics, characterization parameters, and relevant observations associated with the synthesized catalyst.

**TABLE 3:** LCI results of iron-based biomass catalyst synthesis processes.

Impact category	unit	AC preparation	CP	Total
Global warming	kg CO_2_ eq	6.23E+00	6.12E + 00	1.235E + 01
Human toxicity	kg 1,4-DB eq	6.37E-03	1.35E-02	1.98E-02
Photochemical oxidation	kg C_2_H_4_ eq	4.89E-03	5.17E-03	1.006E-02
Eutrophication	kg PO_4---_ eq	6.90E-03	1.47E-03	8.37E-03
Acidification	kg SO_2_ eq	2.65E-03	5.64E-03	8.29E-03

AC, Activated carbon, CP, Catalyst preparation.

The synthesis process of iron-based biomass supported catalyst yields a total impact score in terms of GWP of 1.235E + 01 kg CO_2_ equivalent, as indicated in Table 2. Within this process, the AC stage contributes 52% to the overall GWP, while the preparation stage for the catalyst precursor contributes 48%, as depicted in Figure 4.

Among the chosen impact categories, the GWP exhibits the highest level of impact, primarily attributed to the electricity consumption and the utilization of the activated agent (H_3_PO_4_). This phenomenon specifically pertains to the emission of greenhouse gases during the entire fuel cycle associated with the usage of natural gas for electricity generation. The employment of natural gas in electricity production leads to the release of significant quantities of methane, thereby contributing to the aggravation of global warming. Notably, a considerable portion of the electricity generation in this study relied on natural gas, consequently resulting in the aforementioned detrimental consequences. To effectively mitigate and alleviate these adverse effects, it is imperative to prioritize the adoption and integration of renewable energy sources.

The comprehensive evaluation of the iron-based biomass supported catalyst’s impact score in terms of human toxicity reveals a total score of 1.98E-02 kg 1,4-dichlorobenzene (1,4-DB) equivalent, as indicated in Table 2. In terms of the percentage contributions, the AC preparation stage accounts for 32.5% of the overall impact, while the CP stage contributes 67.5%. The utilization of the activation agent H_3_PO_4_, iron nitrate, and potassium carbonate has been identified as the primary causative factor for human toxicity. Additionally, the employment of electricity during laboratory setups also exerts a significant influence on the human toxicity category. However, it is crucial to acknowledge that the contribution of the activation agent is relatively minor, accounting for approximately 35%, whereas the contributions of iron nitrate and potassium carbonate are substantial, constituting approximately 55% of the total impact. On the other hand, the production of filter paper, waste emissions, and the disposal of waste paper exhibit the least impact on human toxicity. These factors exert a comparatively negligible influence on the overall level of human toxicity when compared to the aforementioned agents.

Photochemical oxidation is a consequential manifestation of air pollution occurring within the troposphere, predominantly induced by the interaction of solar radiation with emissions stemming from the combustion of fossil fuels. This interaction leads to the production of various chemical compounds, including ozone. The deleterious effects of photochemical oxidation encompass respiratory ailments, ocular irritation, as well as harm to both materials and crops. The cumulative impact assessment score for this particular category is quantified as 1.006E-02 kg C_2_H_4_ eq., as shown in Table 3. Notably, the process of AC preparation stage contributes to 47.2% of the total impact, while CP accounts for the remaining 52.8%, as presented in Figure 4. The consumption of power, H_3_PO_4_, iron nitrate, and potassium carbonate in the AC-H_3_PO_4_ and CP processes plays a significant role in influencing photochemical oxidation. In the context of this investigation, a substantial proportion of the electricity utilized was derived from the combustion of fossil fuels. Consequently, the combustion process resulted in the generation of acid pollutants, specifically sulfur oxides (SOx) and nitrogen oxides (NOx).

Eutrophication refers to the progressive augmentation in the levels of phosphorus, nitrogen, and other essential nutrients for plant growth within a maturing aquatic ecosystem. On the other hand, acidification potential pertains to the substances that serve as precursors for the occurrence of acid rain. This category encompasses compounds such as sulfur dioxide (SO_2_), nitrogen oxides (NOx), nitrogen monoxide (NO), nitrogen dioxide (N_2_O), and various other substances. The cumulative impact score and percentage contribution of these impact categories are listed in Table 3; Figure 4. The effect of eutrophication is large during AC production stage due to the utilization of H_3_PO_4_ and electricity while acidification impact dominates in CP due to use of compounds such as iron nitrate, potassium carbonate as well as electricity. It is important to note that largest environmental impacts from consumption of electricity and activation agent utilization apply to all of the considered impact categories. Therefore, it is of utmost importance to thoroughly assess and monitor the levels of activation agent utilization while simultaneously prioritizing the transition from conventional energy sources to renewable ones. This strategic shift holds paramount significance in effectively diminishing the comprehensive environmental footprint linked to all the considered impact categories.

Although various studies have highlighted advancements in catalytic technologies for converting biomass into biofuels (Elliott, 2007)–(Wang et al., 2013), there is limited number of studies on the environmental impacts associated with the use of catalysts in biofuel production processes. For example, the study conducted by Snowden-Swan et al. (Snowden-Swan et al., 2016) provided a life-cycle analysis (from cradle to gate) for the production of two specific catalysts (NiMo/Al_2_O_3_ and Ru/C) and their environmental impacts when applied to the hydrotreating process of fast pyrolysis bio-oil. Their findings suggest that catalyst-related GHG emissions during the conversion process could account for 0.5%–5% of total emissions, varying with the method used for treating co-products. Nevertheless, the authors did not perform a comprehensive LCA of the end renewable hydrocarbon fuel, thus did not estimate on how these catalysts affect the overall life-cycle GHG emissions of the fuel. Similarly, Jones et al. (Jones et al., 2013) found that the impact of catalyst use on the GHG emissions of a biorefinery was minimal, contributing less than 1%. One primary reason that the effect of catalysts on the environmental assessment of biofuel production has not been extensively studied is due to absence of comprehensive data on the materials and energy required for their production, recycling, and recovery. Additionally, details regarding the amount of catalyst used, operational lifespans and exact formulations are often kept as proprietary information.

### 3.2 Study limitations

In this study, a comprehensive LCA was conducted utilizing a life cycle inventory that incorporated predictive data. The evaluation encompassed a combination of analytical determination and experimental measurements to quantitatively assess resource utilization and emissions, disregarding the consideration of uncertainties. However, it is crucial to recognize that this simplified analysis overlooks the crucial aspect of uncertainty, which holds significant importance in substantiating conclusions derived from LCA outcomes (UNEP, 2011). Effectively managing uncertainty within the framework of LCA is of paramount significance, given its utilization of a probabilistic methodology. Various studies investigating the same production chain may yield conflicting conclusions, thereby impeding optimal decision-making processes (Hong et al., 2010), (Huijbregts et al., 2003). To address this challenge, the inclusion of stochastic analysis becomes imperative when undertaking an LCA that includes uncertainty assessment. Commonly employed techniques include Latin hypercube and Monte Carlo sampling, necessitating the utilization of probability distributions for critical life cycle variables, including energy consumption, chemical reagents, yield, flow, emissions, and adsorption capacity.

Nevertheless, incorporating uncertainty factors into LCA for catalyst synthesis poses certain challenges. This can be primarily due to the scarcity of information regarding the probability distribution of these parameters within the existing body of literature. Consequently, a notable knowledge gap exists within this specific domain, thus presenting a promising opportunity for future research studies and advancements.

Moreover, the paper’s limitations primarily revolve around the scope of environmental impact categories assessed, reliance on specific geographic data which may not be universally applicable, and the exclusion of economic and social considerations such as labor costs and safety protocols. For improvement and broader applicability, future studies could expand environmental impact categories, incorporate a global perspective with diverse geographic data, and include economic and social factors for a more holistic sustainability assessment. This approach would enable the methodology to be adapted for different industrial processes, enhancing its utility across various sectors.

### 3.3 Economic analysis

#### 3.3.1 Catalyst production cost

The cost of the biomass supported catalyst produced from waste biomass relates to the cost of waste biomass collection as well as the cost of several preparation phases such as cleaning, drying, chemical purchases, and carbonization (the cost of the power necessary to heat the furnace). Because the waste biomass in lab scale study projects it comes from the university’s or residential areas, the only costs examined for the lab-scale production of biomass supported catalyst were those associated with the usage of chemicals and power. The chemicals and electrical expenses were between 1.22 and 1.50 $ per kg.

#### 3.3.2 Catalyst testing cost

The catalyst testing cost is associated with the cost of gases (H_2_, CO_2_, CO, and N_2_) and the cost of the heating supply during the reaction carried out in the fixed bed reactor. The product composition was analyzed through GC and GC-MS machines. The GC and GC-MS electric costs, as well as the gases required to operate the machines, are associated with the cost of catalyst testing. The lab-scale catalyst and their product analysis cost in the range of 8.2–10.50 $ per kg.

## 4 Conclusion

The comprehensive LCA of iron-based biomass catalyst synthesis processes was performed across various environmental impacts categories. The study findings reveal that GWP and human toxicity are two of the most significant environmental effects, with both being largely influenced by electricity consumption and the utilization of specific reagents. Electricity consumption, predominantly derived from natural gas, emerges as a significant contributor to GWP. These findings highlight the imperative need to shift towards renewable energy sources to mitigate greenhouse gas emissions and, consequently, global warming effects. The activation agent H_3_PO_4_, iron nitrate, and potassium carbonate significantly influence human toxicity, with the latter two agents accounting for a substantial 55% of the total impact. Photochemical oxidation, a consequence of air pollution, eutrophication and acidification effects further highlight the environmental implications of the synthesis processes, pointing towards the need for resource optimization and a shift in energy consumption patterns. Interestingly, while this study offers valuable insights into the environmental consequences of the iron-based biomass catalyst synthesis processes, the simplified nature of our analysis, which excludes uncertainties, is a limitation. Addressing uncertainties in LCA is crucial, and future studies should endeavor to incorporate probabilistic methodologies to ensure a more comprehensive and robust assessment. This will enable better decision-making processes, especially when varying studies present contradictory conclusions. The scarcity of information on probability distribution parameters in current literature highlights a significant knowledge gap, presenting a ripe avenue for future research. Furthermore, future studies should consider system boundaries expansion incorporating both utilization and end-of-life phases. While this study was performed in a controlled laboratory environment, there is a pressing need to examine catalyst production methodologies in large scale industrial settings. The industrial fabrication of iron-based biomass catalysts would employ varied equipment and potentially exhibit distinct environmental consequences.

## Data Availability

Upon legitimate request and in accordance with regulations, the underlying data that substantiates the findings in this article will be provided.
